# Case Series: Free vascularized costochondral grafts in upper extremity reconstruction

**DOI:** 10.3389/fsurg.2025.1478253

**Published:** 2025-02-25

**Authors:** Saskia J. M. Kamphuis, Dirk J. Schaefer, Alexandre Kaempfen

**Affiliations:** Department of Plastic, Reconstructive, Aesthetic and Hand Surgery, University Hospital Basel, Basel, Switzerland

**Keywords:** reconstruction, autologous, cartilage, defect, rib graft, case series

## Abstract

**Introduction:**

Costochondral grafting is well-known reconstructive option for the temporomandibular joint. In the upper extremity, non-vascularized costochondral grafts have been used for radial head reconstruction, for osteoarthritis of the trapeziometacarpal joint and proximal pole reconstruction of the scaphoid. Evidence suggests that vascularization of bone grafts lead to a higher union rate and a faster time to union. To avoid other donor sites and improve healing times we endeavored using vascularized costochondral grafts for skeletal reconstruction of the hand.

**Methods:**

In this report, we present the operative technique and three cases of reconstructions. They comprise one case of a third metacarpal head defect including cartilaginous tissue due to avascular necrosis (Mauclair's or Dieterich's disease), one case of scaphoid proximal pole reconstruction due to necrosis and one case of a third metacarpal head reconstruction after direct trauma. Patients’ complaints included pain and clicking of the joint upon movement of the joint. There were also concerns about osteoarthritis and joint destruction on the future. Medium-term outcome showed good results concerning pain as well as normal range of motion without clicking of the joint.

**Conclusion:**

Osseocartilaginous grafts are not readily available as a reconstructive option. Donor sites at the knee, metatarsophalangeal and the hamate risk a permanent damage and functional deficit. Furthermore, these grafts are not easily vascularized. Costochondral grafts present a viable option with a challenging dissection, but no mid- to long-term functional loss at the donor site.

## Introduction

Costochondral grafts are widely used non-vascularized for the treatment of craniofacial defects, especially the reconstruction of the temporomandibular joint, concha, nose, and trachea (1, 2). Larger bone defects benefit from reconstruction with vascularized bone grafts as bony healing is improved both in quality and time. The same applies to bone healing in poorly vascularized tissue environment or infection (3).

Due to its reliability and rapid reestablishment of skeletal stability which enables earlier rehabilitation of motion, vascularized bone grafting seems especially beneficial in the upper extremity (4). A spectrum of other treatment options for promotion of boney healing in the upper extremity have been proposed in the literature. These options range from local pedicled flaps to non-vascularized distant grafts and other vessels to promote bone healing like platelet-rich plasma (5–7). The results of all techniques vary strongly and are dependent on multiple factors, including surgeon experience and -preferences.

Non-vascularized use of costochondral grafts in the upper extremity has been described for osteoarthritis of the trapeziometacarpal joint, reconstruction of the radial head and the scaphoid (8–10). Application of a vascularized costochondral graft for reconstruction in the upper extremity was described only scarcely for reconstruction of the scaphoid and an ulnar defect (11–13).

We detail the technique of vascularized costochondral graft harvest, its indication for defect reconstruction and outcomes demonstrated by three cases.

## Operative technique

### Anatomical considerations

Preoperative localization of the osseocartilaginous junction is feasible by clinical palpation of anatomical landmarks or by ultrasound. The osseocartilaginous connection is found on the milk line which runs from the middle of the clavicula over the nipple-areola-complex further caudally. Here a developmental stop of endochondral ossification takes place in anatomical areas that need biological flexibility, for example for breathing movements in the ribs. This process guarantees a preservation of cartilage (14). Therefore, we prefer ribs five to seven as their cartilage remains flexible during life.

One should consider the curve and the location of the vessel according to the needs of the donor side when choosing either the ipsi- or contralateral rib. If two surgical teams are planned to prepare the recipient site and graft extraction simultaneously to shorten operating time, the contralateral harvest side is preferred to facilitate staff placement in the operating theatre.

### Donor site preparation

**Figure 1:**
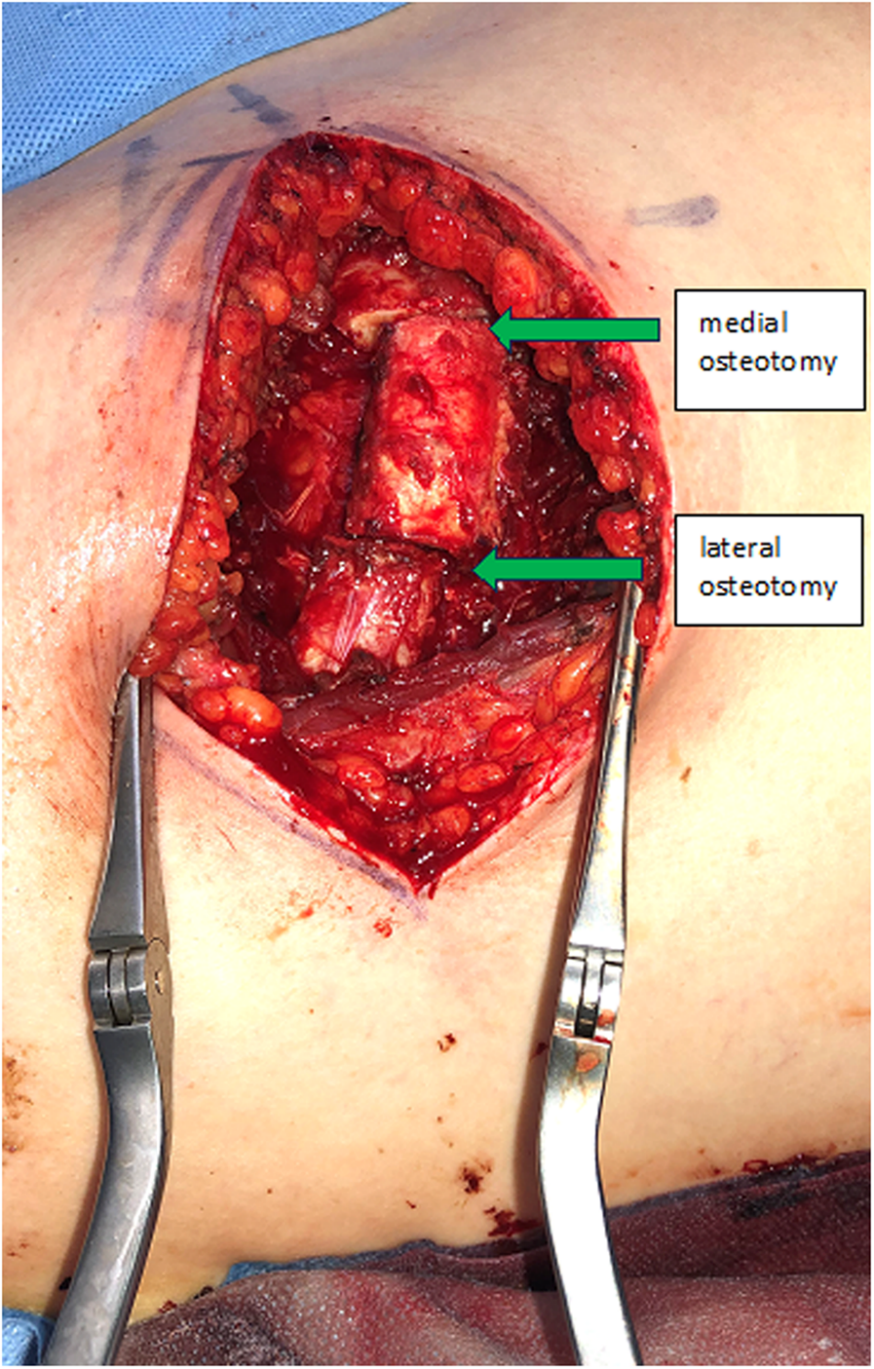
Donor site of the costochondral graft of the fifth right rib with osteotomy levels.

**Figure 2:**
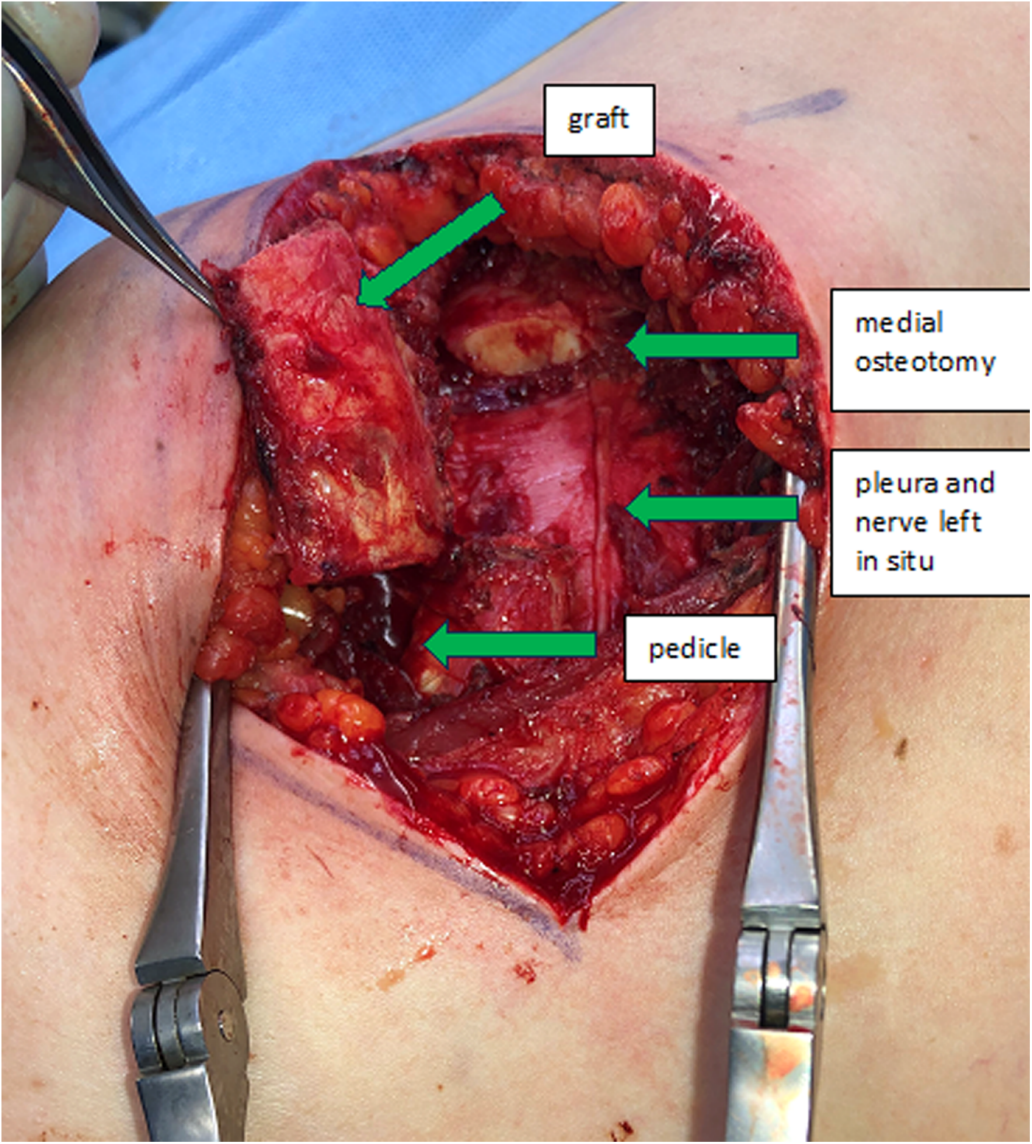
Donor site of the precut costochondral graft of the fifth right rib including pedicle.

The incision follows the curve of the chosen rib. In woman the submammary fold can be used. The thoracic wall is exposed by detaching the rectus abdominis muscle from the ribs. In caudal ribs a muscular transection is sometimes beneficial for reconstruction after the harvest. Subsequent steps involve exposing the costochondral junction and detaching the cranial and caudal intercostal muscles, protecting the neurovascular bundle underlying the caudal aspect of the rib and the periosteum in the anterior aspect of the rib. The rib is then cut medially to the osseocartilaginous junction through cartilage with a sharp blade, preserving enough distance to allow mobilization of the graft and modelling to the desired shape (Figure 1). Now the graft can be elevated off the pleural plane by blunt dissection, using a periosteal elevator. Preservation of the periosteum on the graft is desirable, but not always possible due to rigid connections to the pleura, which should be preserved to prevent a pneumothorax. At this stage, the graft is liberated also on its bony connection lateral to the osseocartilaginous junction. It is vital to identify and protect the neurovascular pedicle and the pleura when using an oscillating saw or an osteotome. Now the graft is only held in place by its pedicle (Figure 2). The medial or lateral pedicle can be chosen depending on the size of the vessel and anatomical requirement at the recipient site. Sometimes only small choke vessels between the anterior vessels coming off the internal mammary and the lateral costal arteries mark the osseocartilaginous junction and the periosteum and perichondrium must be preserved in this area of the graft to ensure vascularization. Short grafts are therefore at risk of losing vascularization during final stages of exposure and trimming.

Finally, the nerve is separated from the costal artery and its accompanying (possibly small or absent) vein. Dissecting long pedicles is cumbersome in between and underneath the remaining rib, but several centimeters can be dissected on either side to reach the desired length. The flap is fully detached and transferred to the recipient site.

Before closure of the donor site, a Valsalva maneuver is performed to detect any pleural defects, which are then meticulously patched using adjacent musculature or fascia. The rib defect is reconstructed using a Vicryl mesh pouch filled with diced cartilage and bone fragments from flap trimming, placed within the defect of the original rib. To minimize pain, the costal nerve is blocked with local anesthetic and the wound is closed in layers over a drainage.

### Recipient site preparation

Costal arteries are small, matching recipient vessels on the hand include the first dorsal intermetacarpal artery for metacarpal reconstructions or the palmar branch of the radial artery for scaphoid reconstructions, among other available options. Shaping and tailoring of the graft to suit the defect contours is performed using a knife or bone nibblers. Fixing the graft in position is performed using screws, plates, or stitches, depending on the location. The pedicle and mentioned choke vessels must be considered during positioning and fixation. In our experience, we could reach a pedicle length of four to five centimeters, depending in the needed graft size. In case a longer pedicle is needed, an extension to the internal mammary artery is an option.

Supramicrosurgical anastomosis ensures arterial perfusion. We perform the anastomosis under an operation microscope magnification by a surgeon with experience in microsurgery in level III, IV or V (15). Venous drainage is sometimes ensured by bleeding only, as the donor vein is often too small or completely missing. Therefore, a wound drainage is mandatory for three to five days, allowing time for vessel inosculation.

Affected joints were stabilized by splinting in our case series, depending in the involved joint. Once a computer tomography (CT) scan confirmed at least 50% osseous healing of the bone cross-sectional area, range of motion exercises were started, guided by occupational therapists.

## Case descriptions

**Figure 3:**
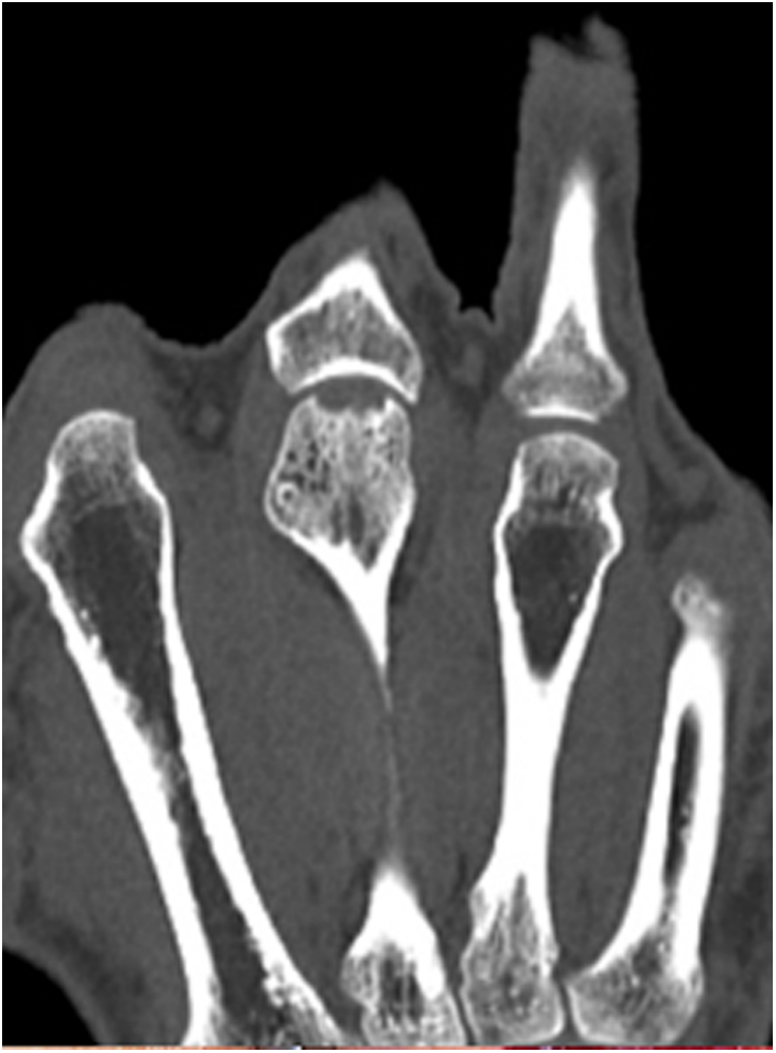
Case 1 defect of the metacarpal head and its cartilage of 7 by 4 mm with 2 mm joint depression.

**Figure 4:**
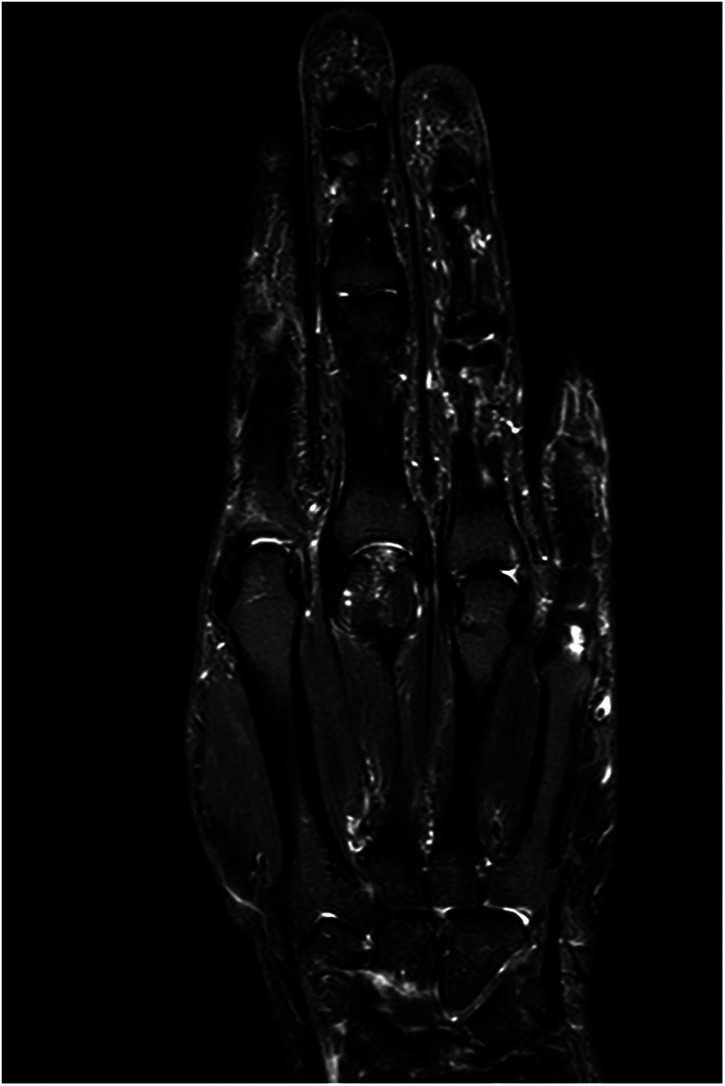
Case 1 corresponding contrast-enhanced MRI image to Figure 3 with signs of avascular necrosis.

**Figure 5:**
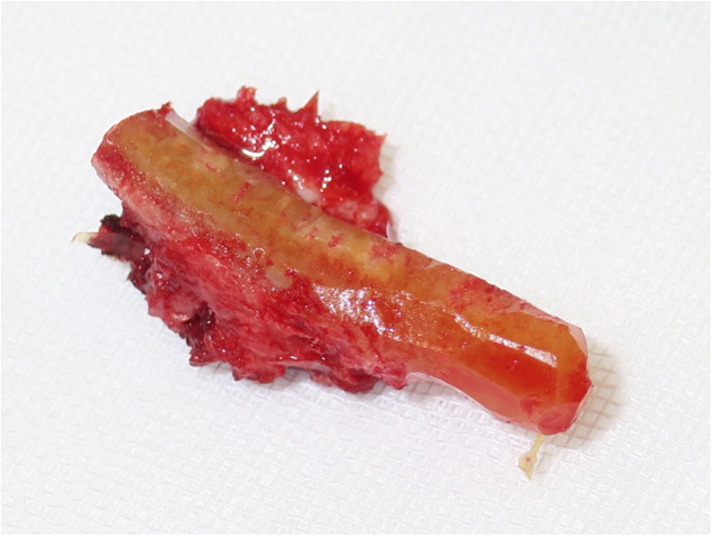
Graft formation with muscle cuff, pedicle not visible in this image.

**Figure 6:**
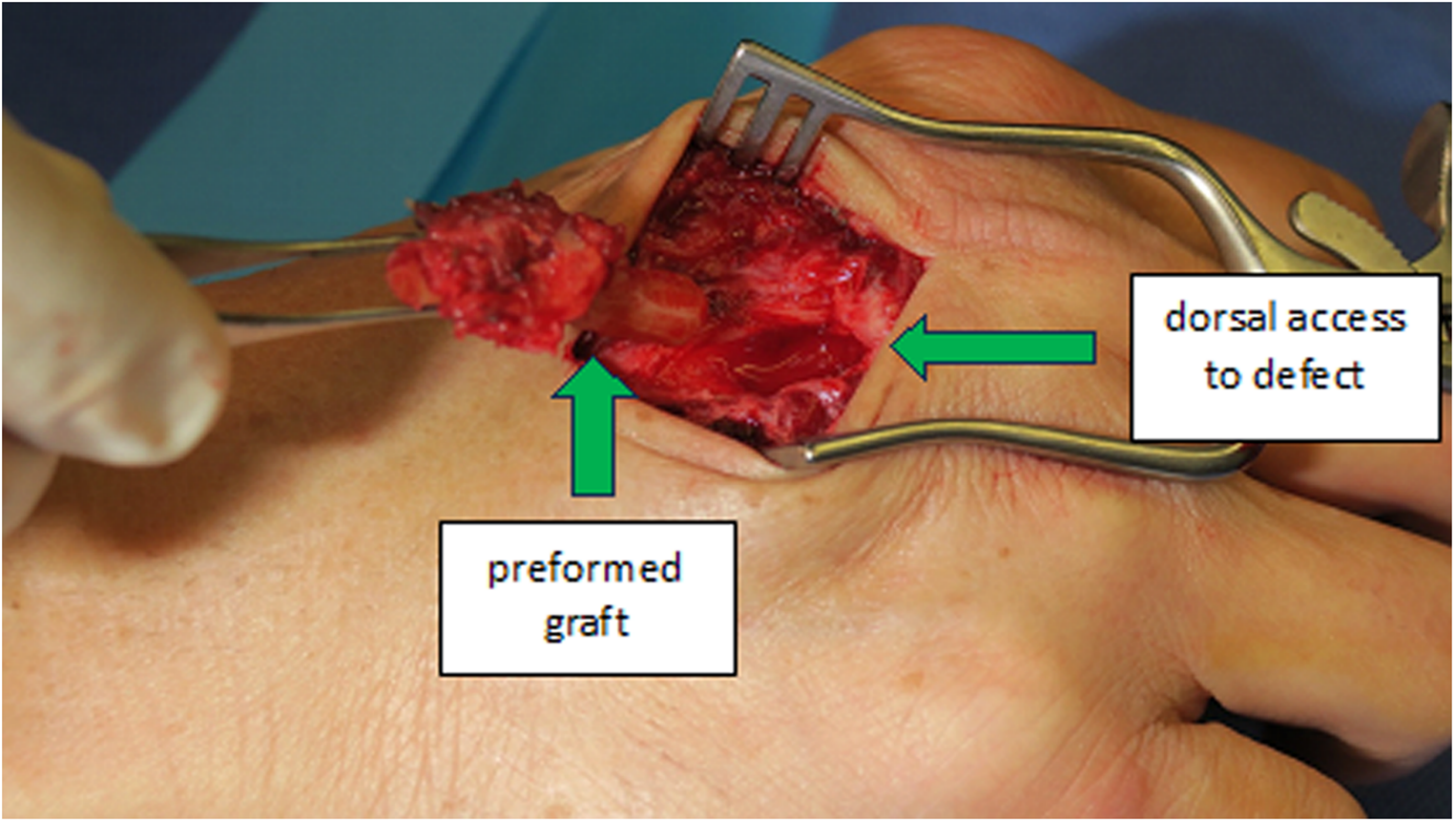
Case 1 inset of the shaped costochondral graft through a dorsal tunnel to the defect.

**Figure 7:**
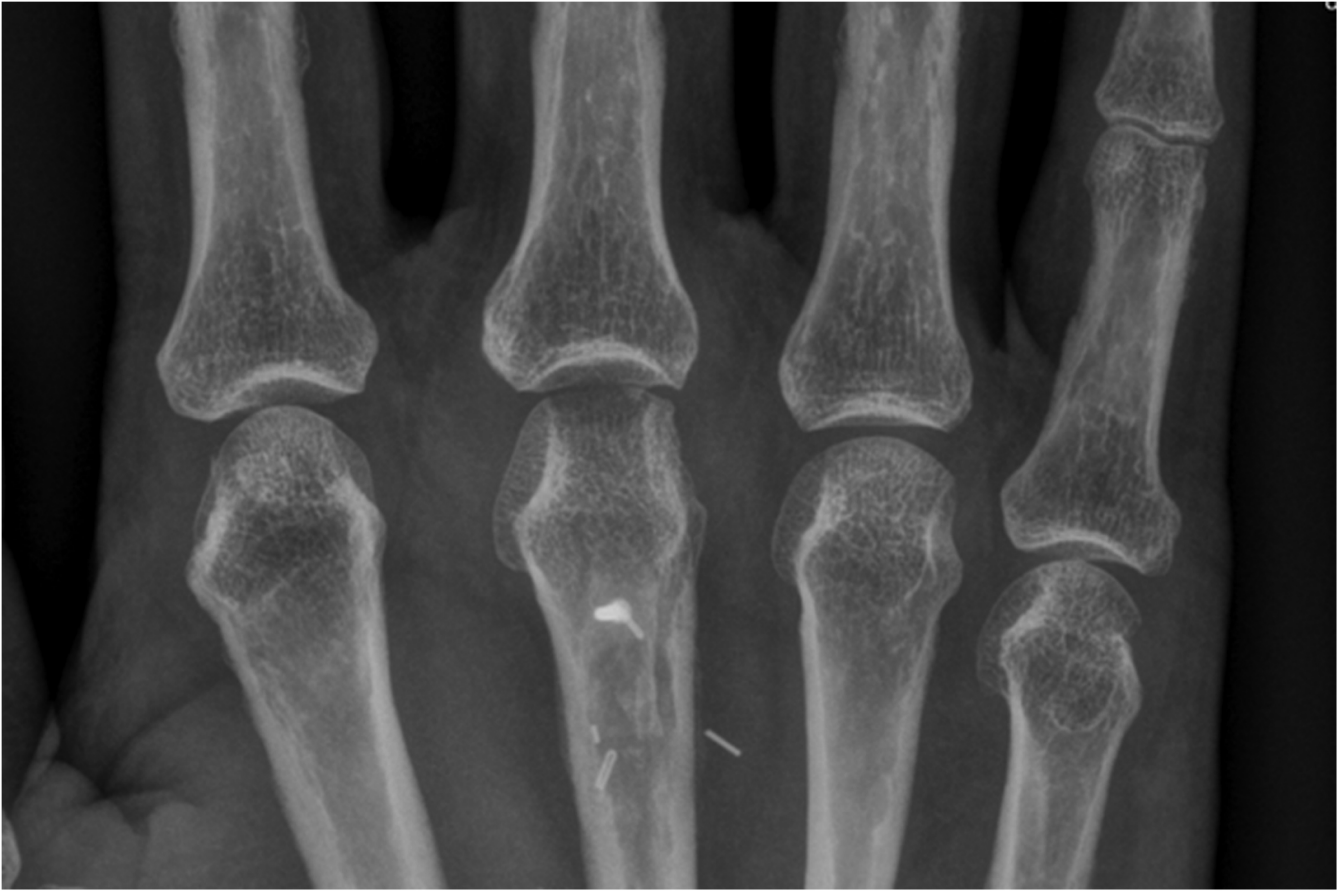
Case 1 postoperative result after rehabilitation.

Our first case concerns a 62-year-old physically very active male, who presented with pain and restricted range of motion in the third metacarpophalangeal (MCP) joint due to a cartilage defect linked to Mauclair's or Dieterich's disease, a relatively uncommon occurrence of avascular necrosis affecting the metacarpal head (16, 17). The patient was specifically bothered by the inability to perform a full fist closure. Previous cancellous bone grafting in the metacarpal head yielded unsatisfactory outcomes. Upon evaluation in our clinic, a CT and magnetic resonance imaging (MRI) revealed a cartilaginous metacarpal head defect measuring seven by four millimeters, with a two-millimeter joint depression, while the cartilage of the base of the proximal phalanx was deemed adequate (Figures 3 and 4). We discussed various treatment possibilities with the patient, who preferred non-prosthetic options. Considering non-vascularized bone grafting had previously proven ineffective for pain relief in this case, we decided together with the patient to perform an autologous reconstruction involving a contralateral free vascularized costochondral graft. The graft was prepared as outlined above. It included a lateral pedicle and was inserted into the metacarpal head through a tunnel, drilled from the dorsal metacarpal shaft to the defect, which was planned preoperatively guided by the CT imaging (Figures 5 and 6). The refinement of the graft was accomplished by arthroscopically, by shaving the cartilaginous segment. The microsurgical anastomosis was conducted from the intercostal artery to the intermetacarpal artery of the second commissure. The patient exhibited a remarkable recovery, returning to his regular activities including his professional capacity in a supervisory role and golfing in just three months. A subsequent 16-month follow-up revealed the patient's satisfaction with minimal residual stiffness during forceful hyperextension and power grip. Despite a 10 degrees flexion deficit in the MCP joint, the patient enjoyed uninhibited range of motion and full fist closure. Notably, grip strength on both hands measured 50 kilograms, while pinch strength on the affected digit stood at four kilograms, compared to six kilograms on the contralateral side. No signs of osteoarthritis were noted in the follow up x-rays on which the graft was clearly visible without ossifications at one year follow-up (Figure 7).

Our second case concerns a 38-year-old male had a history of a proximal scaphoid fracture three years prior, treated with percutaneous screw fixation. Unfortunately, non-union development and loosening of the screw prompted intervention. After removal of the screw and arthroscopic debridement of cysts, transient relief was achieved. However, upon resuming his usual activities as a laboratory device installer, pain complaints resurfaced due to a proximal pole ischemia, which was confirmed by MRI. The patient was informed about various reconstructive alternatives, including a medial femur condyle osseocartilaginous graft, partial arthrodesis and prosthetic options. Given his age and active lifestyle, primary arthrodesis and prosthesis were ruled out. Moreover, the patient declined a medial femur condyle graft due to concerns about potential knee pain as a passionate recreational soccer player. Consequently, the option of reconstructing the proximal scaphoid with a contralateral free vascularized costochondral graft was discussed and agreed upon. The procedure entailed utilizing a vascularized costochondral graft harvested from the fifth rib on the right side harvested on the medial pedicle, fixated to the scaphoid of the contralateral left wrist using two Kirschner wires and a Linscheidt wire allowing for the scapho-lunate (SL) interosseous ligament to heal after reattachment. The SL ligament could be fixated on the graft by a remaining flake on the ligament. A microsurgical anastomosis between the intercostal artery and the palmar branch of the radial artery was accomplished despite minimal mismatch. Postoperatively, the Kirschner wires were removed at the three-month mark after progressive bony healing, and mobilization was initiated. However, rehabilitation highlighted the graft's size discrepancy, which was corrected through arthroscopic shaving. This adjustment ultimately led to an acceptable range of motion—30 degrees of extension and 40 degrees of flexion. The patient successfully returned to an adapted occupational role after five months. Long-term follow-up showed good integration and stability of the costochondral graft despite a new fall and a first metacarpal fracture two-and-a-half years postoperatively, which could be treated non-operatively.

**Figure 8:**
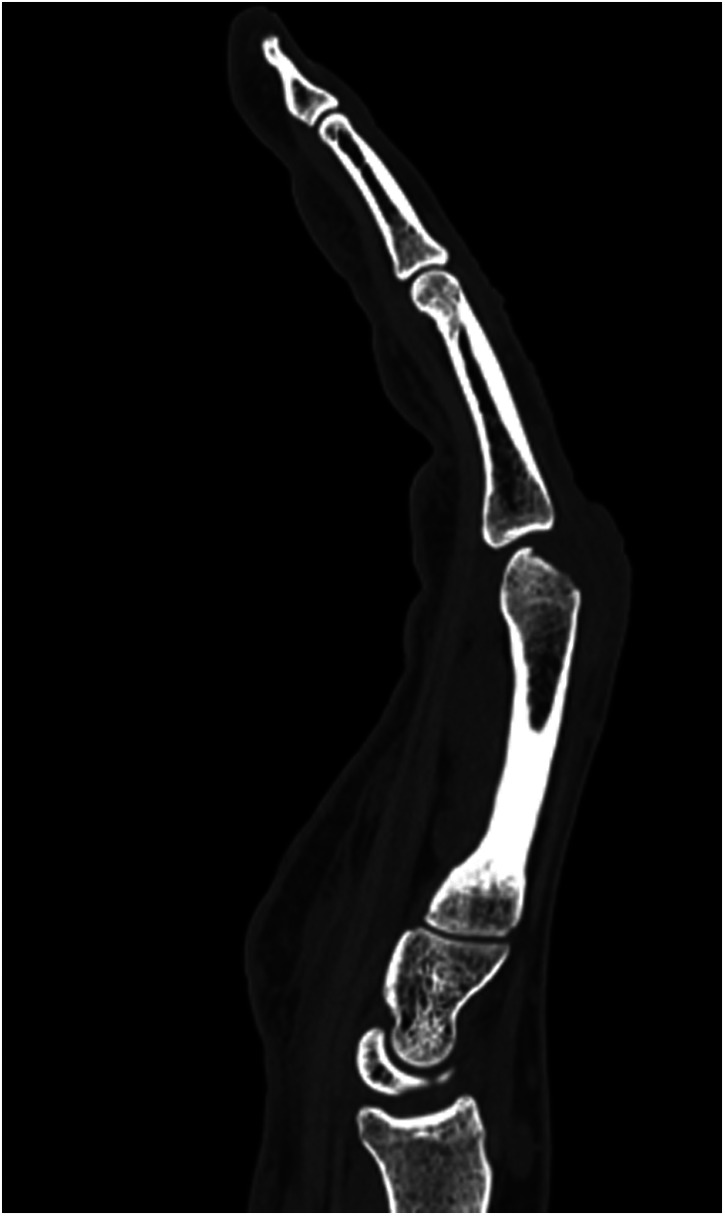
Case 3 preoperative CT imaging of dorsal defect of the third metacarpal head.

**Figure 9:**
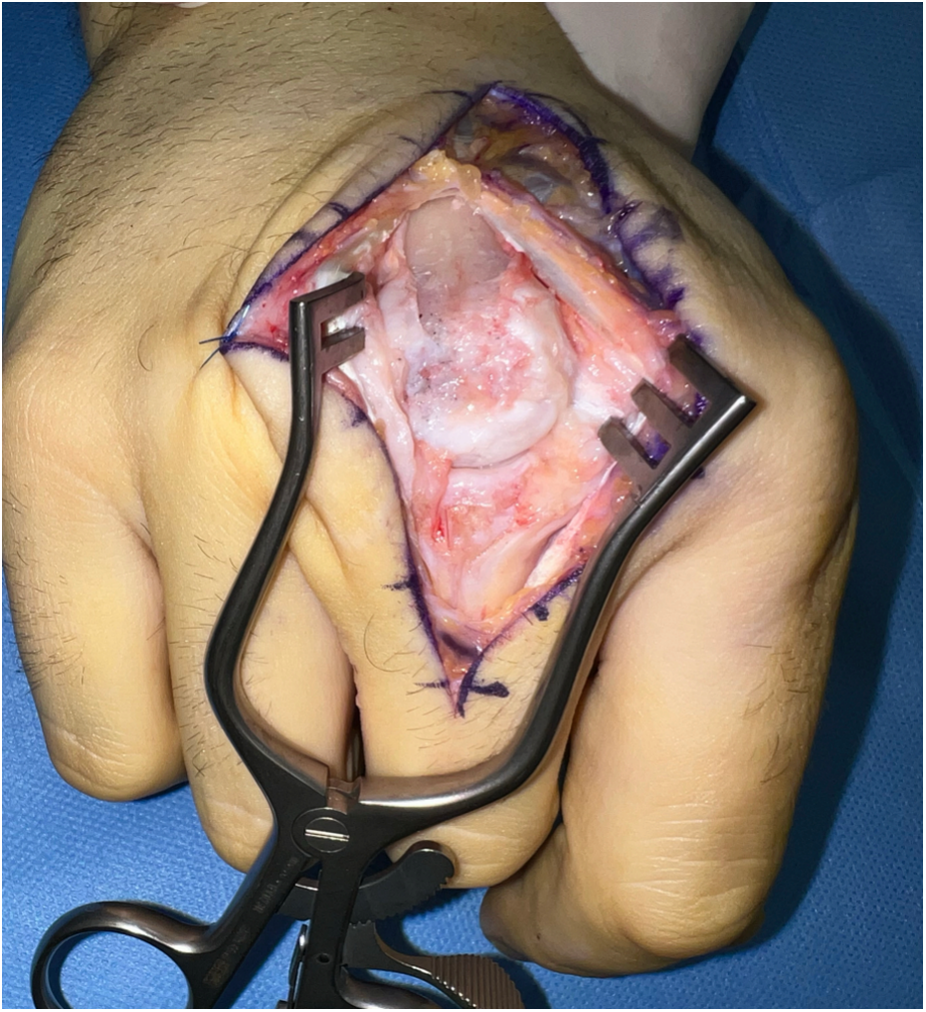
Case 3 intraoperative visualization of the chondral defect of the third metacarpal head.

**Figure 10:**
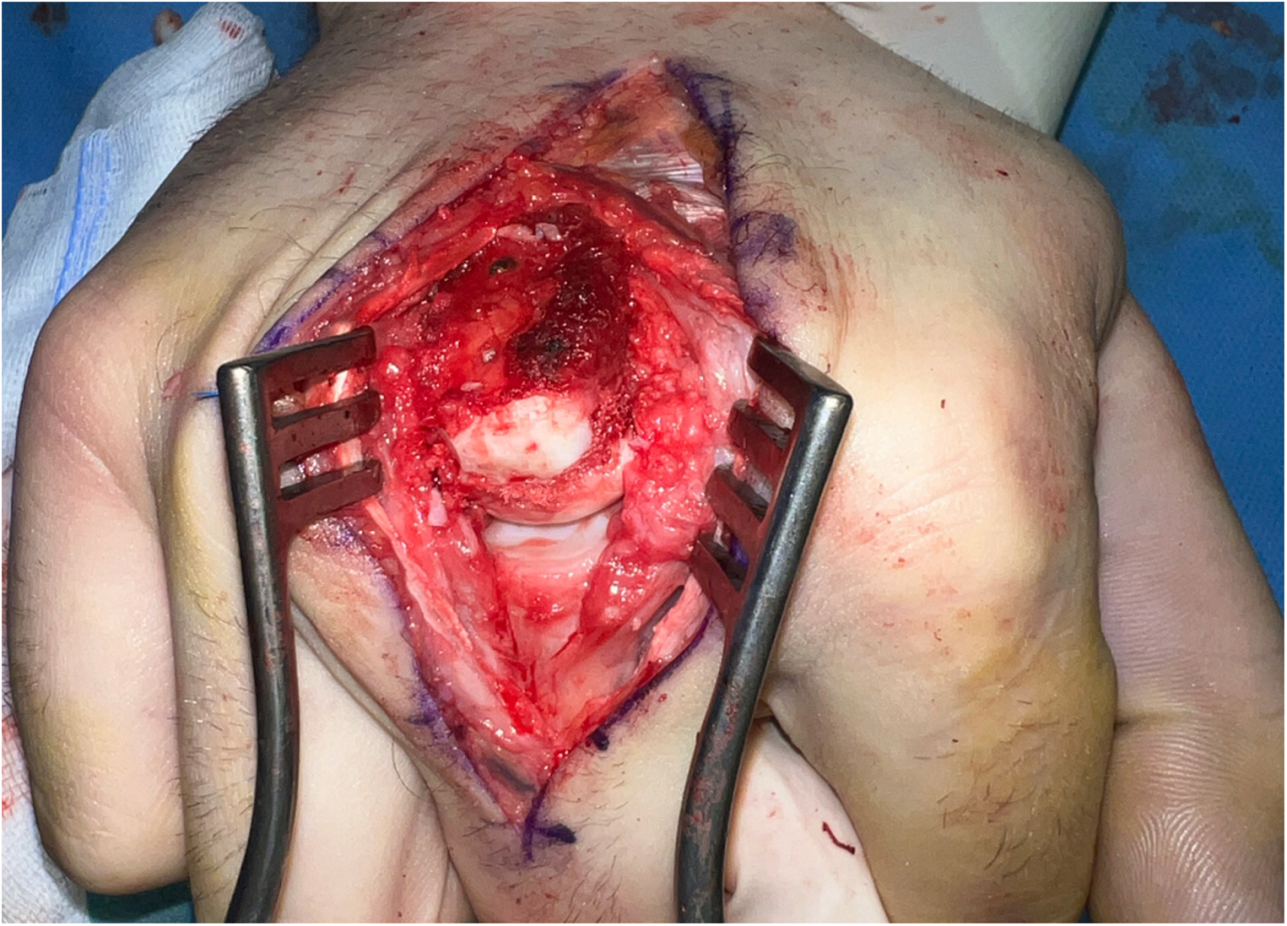
Case 3 intraoperative visualization of the reconstructed chondral surface of the third metacarpal head.

**Figure 11:**
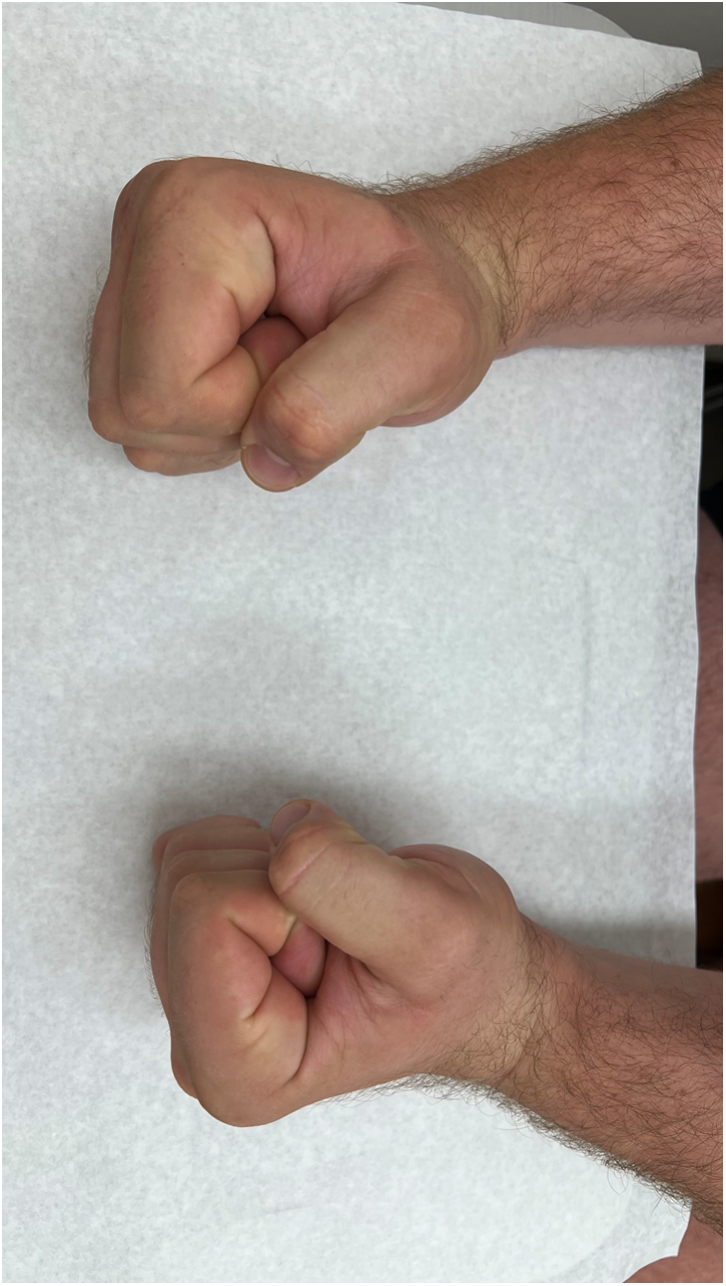
Clinical image of fist formation of both hands (right hand treated, left hand comparison).

**Figure 12:**
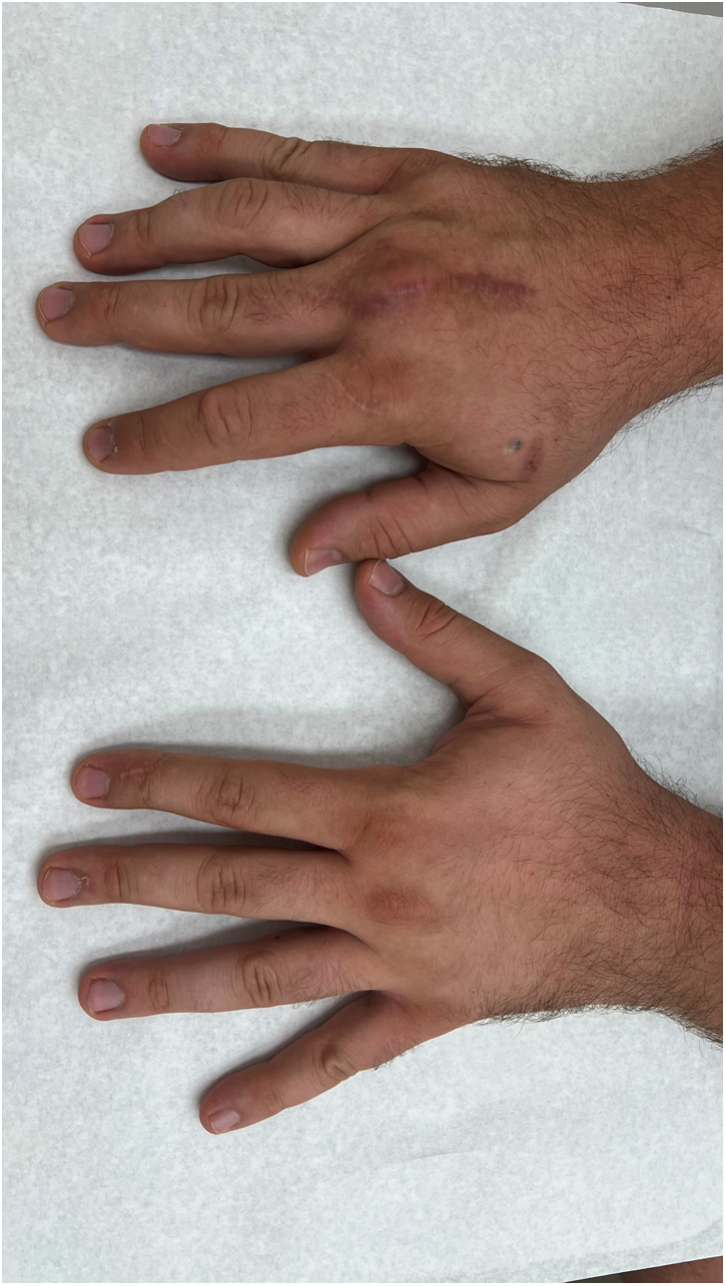
Clinical image of extension of all fingers (right hand treated, left hand comparison).

In our third case a 23-year-old male sustained a dorsal impression fracture of the third metacarpal head when attempting to strike a boxing ball in a festival machine (Figure 8). Due to his young age and physically demanding job on a building site, the patients’ main concern was pain and future osteoarthritis. Initial treatment was non-operative, with a subsequent improvement in active range of motion as swelling subsided. However, a painful clicking sensation emerged during joint movement. MRI confirmed intact base cartilage of the proximal phalanx. Considering these findings, a decision was made to reconstruct the metacarpal head using a contralateral free vascularized costochondral graft. The defect was visualized during the operation (Figure 9), after which, the graft was anchored to the remaining metacarpal using one-and-a-half millimeter cortical screws (Figure 10), with a microsurgical anastomosis linking the lateral intercostal artery to the second dorsal intermetacarpal artery, both of similar caliber. Postoperative management included initial cast immobilization, transitioning to a relative motion splint after two weeks. During the first phase of rehabilitation, a slight clicking of the joint occurred, which also disappeared spontaneously after further mobilization under guidance of the occupational therapists. Range of motion and strength is satisfactory (Figures 11 and 12), but due to his age the patient decided to pursue further education with the goal of obtaining a less physically demanding occupation in the future.

## Discussion

Our case series demonstrates good results of the free vascularized costochondral graft technique for reconstructions in upper extremity surgery. All patients could rehabilitate to satisfaction and reintegrate in daily personal and work environment. Although the range of motion reached was not completely normalized, none of the patients were hindered in daily life. In addition, all patients could reach a stable state of functionality of the hand without any pain. Subjectively, all patients were very satisfied with the results and would undergo the same procedure in the same situation again. Costochondral bone healing was uneventful and rapid in all cases, despite challenging environment of the osteosynthesis.

Most research in vascularized reconstructions in hand surgery have been performed on scaphoid non-union cases and show benefits from vascularized grafts (18–20). Larger osseocartilaginous grafts are usually harvested from the second metatarsal phalangeal (MTP) joint or from the medial femur condyle (MFC), smaller grafts can also be harvested from the hamate. Disadvantages include potential functional donor site morbidity with reduced weight bearing capacity and/or reduced strength as well as persisting complaints and the possibility of premature osteoarthritis. In the literature, donor site complication rates for MFC grafts of 18% are described (21), and for the MTP joint graft, floppy and unstable toes with visible deformities have been described in up to 100% of cases (22). The expected range of motion after reconstructions using these techniques is described between half and two-thirds of the normal range of motion, depending on the anatomical location of the reconstruction. In comparison to these donor sites, the advantage of the costochondral graft would be the hidden scar, in women even in the inframammary fold. The disadvantage is the risk of intraoperative respiratory complications like a pneumothorax and postoperative breathing discomfort and/or development of pneumonia. The symptoms postoperatively are comparable to those of a fractured rib. However, after six weeks, none of our male patients reported any persisting complaints concerning the respiratory tract and none had complications such as a pneumothorax or pneumonia. None was concerned by the aesthetics of the scar on the chest.

Our series show the results of two patients with a metacarpal head reconstruction and one with a scaphoid reconstruction. Both patients with the reconstructions of the metacarpal head were treated using a relative motion splint after wound healing. This facilitated early mobilization to prevent full stiffness after bone consolidation. This strategy was successful in that, after three months both patients reached a full fist closure and were very satisfied with comfort during the rehabilitation period. The patient with the scaphoid reconstruction was immobilized using a plaster cast until one consolidation, due to the healing process of the SL ligament and the wrist dynamics.

Different techniques and levels of rib cartilage donor sites have been described, for example, the fifth, the sixth rib (9, 12) and the seventh rib (23). We prefer the fifth rib due to a good cartilaginous part and an easier approach at the cranial end of the rectus abdominis muscle.

After transplanting a graft from its original position to its new function, the environment changes. The influence of the new vascularization, pressure and movement can lead to changes in density of the graft. Where it started as a chondral graft, it is possible that calcification occurs. This form of ossification consists of a complex sequence of proliferation, differentiation, and tissue remodeling events and comprises multiple distinct steps of late cartilage differentiation. Each of the steps is subject to positive and negative control by environmental signals. Some form of calcification is inevitable, however, care should be taken to prevent full ossification of the graft, as the cartilaginous part is important for joint reconstructions (14, 24). In our series, fortunately, we did not encounter full or any problematic ossification of the graft up until a follow-up of at least one year.

Recently, vascularization is thought to be also important for smaller bone defects, to allow for faster bone healing and motion rehabilitation (4). Moreover, in patients with compromised soft tissues or infection, vascularization is an absolute necessity. Immediate perfusion of the transplanted graft is thought to promote primary bony healing instead of secondary healing, which takes significantly more time and is more prone to delayed- or non-unions.

Despite the positive results from our first experience using this technique, our number are small, which is a limitation in the formation of definitive conclusions. Further research into the technique is warranted to proof reliability. Also, the long-term ossification risk and its prevention should be further investigated. Optimally, a study including a control group using other reconstructive techniques and a correct sample size calculation should be performed. Main objective would be to prevent ongoing osteoarthritis due to joint destruction in young and employed patients, in which case salvage options should be avoided.

## Conclusion

Our case series demonstrates the feasibility of vascularized osseocartilaginous bone grafting from the rib with satisfactory clinical results from the subjective patients’ perspective as well as objective measurements. The results allowed all patients to resume their activities of daily life and to a large extend their employment. Larger case series are warranted to proof the reliability and further potential pitfalls of the technique.

## Data Availability

The original contributions presented in the study are included in the article/Supplementary Material, further inquiries can be directed to the corresponding author.
